# The Selenium Supplementation Influences Olive Tree Production and Oil Stability Against Oxidation and Can Alleviate the Water Deficiency Effects

**DOI:** 10.3389/fpls.2018.01191

**Published:** 2018-08-15

**Authors:** Roberto D’Amato, Mauro De Feudis, Paul E. Hasuoka, Luca Regni, Pablo H. Pacheco, Andrea Onofri, Daniela Businelli, Primo Proietti

**Affiliations:** ^1^DSA3, Department of Agricultural, Food and Environmental Sciences, University of Perugia, Perugia, Italy; ^2^Instituto de Química de San Luis, INQUISAL, Centro Científico-Tecnológico de San Luis (CCT-San Luis), Consejo Nacional de Investigaciones Científicas – Universidad Nacional de San Luis, Chacabuco y Pedernera, San Luis, Argentina

**Keywords:** seleno amino acids, olive oil stability, drought stress, antioxidant compounds, *Olea europaea* L., phenols

## Abstract

Foliar fertilization with selenium (Se) may well be beneficial in increasing the nutritional and qualitative values of food in Se-deficient regions such as the Mediterranean Basin, and may contribute to an increase in drought resistance in plants. The present study has considered detachment force, flesh firmness, pigmentation, fresh and dry weight, and oil content of olive drupes from Se fertilized olive orchards (*Olea europaea* L.) under drought stress and well-watered conditions. This study has also evaluated the total Se, Se amino acid, phenol, carotenoid and chlorophyll contents of EVOO, plus its oxidative stability against oxidation. While there was no change in the ripening indexes and the production of olives generally, Se application did increase the total Se, Se methionine, phenol, and carotenoid and chlorophyll contents. The higher concentration of these (bio) chemical compounds in EVOO obtained from Se fertilized plants might well suggest enhanced antioxidant activity. Consequently, EVOO obtained from Se fertilized trees possesses a higher nutritional value and, as indicated by the greater oxidative stability against oxidation, longer shelf life. In addition, under water deficient conditions, a higher fresh olive weight corresponds to a higher level of phenol, carotenoid and chlorophyll, and the chlorophyll-to-carotenoid ratio in Se fertilized trees would appear to confirm the positive role of selenium in alleviating damage caused by drought stress conditions.

## Introduction

Selenium (Se) is essential for humans since it integrates GPx an enzyme that plays an important role in cerebral and reproductive functions (Navarro-Alarcón and López-Martínez, 2000; Rayman, 2000), prevention of cancer and cardiovascular disease (Rayman, 2000; Roman et al., 2014), and the detoxification of heavy metals. Nutritionists encourage Se-enriched food consumption, especially in areas where soil Se levels are low, which is the case in certain parts of Europe (Sager, 2006). As a result, interest in Se-enrichment in food is steadily increasing (Rayman, 2000; Finley, 2007; Businelli et al., 2015; D’Amato et al., 2017; Fontanella et al., 2017).

Olive oil is a key component in the traditional Mediterranean diet, and the olive tree (*Olea europaea* L.) is one of the oldest and most important tree species in the Mediterranean Basin accounting for 98% of the world’s olive cultivation (Banias et al., 2017). In fact, European countries provide 80% of the olive oil produced worldwide, and Italy is the second largest olive oil producer after Spain (Banias et al., 2017). The importance of EVOO in these regions coupled with a low level of Se in the area (Spadoni et al., 2007) has forced researchers to develop new methods of increasing the Se concentration of olive oil. Previous studies have demonstrated Se enrichment in EVOO obtained from Se fertilized olive trees (Proietti et al., 2013; D’Amato et al., 2014). Once administrated because of its chemical similarity to sulfur (S) Se is generally taken up by the plant via sulfate transporters and then metabolized via the S assimilation pathway (Sors et al., 2005), which may incorporate this micronutrient into organic forms such as SeCys and SeMet. Recently, interest in seleno-amino acids has increased because they integrate the active centers of several selenoenzymes, which are involved in biological synthesis and plant metabolism, and are used for protein structure determinations (Iwaoka et al., 2008). For example, the study performed by Torres et al. (2014) on EVOO obtained from olive groves in Argentina grown on soil containing Se, has shown the presence of seleno-amino acids such as SeMeSeCys and selenocysteine. Data concerning the effect of these Se compounds on EVOO is scarce, but Zalejska-Fiolka (2000) who demonstrated the anti-oxidative properties of α-tocopherol, SeMet and methionine in olive oil, noted greater anti-oxidative properties in SeMet than in α-tocopherol.

A key element to EVOO quality might well be the drought periods that generally occur in the Mediterranean area during the spring and summer periods (Spadoni et al., 2007; Proietti et al., 2013). Indeed, during water limitation, a reduction of net photosynthesis and an increased production of ROS occurs in plants (Kaushik and Roychoudhury, 2014), and this might undermine EVOO stability against oxidation. In this context, Se appears to be involved in the mechanism of water-stress tolerance, via ROS detoxification (Feng et al., 2013). This aspect helps to preserve membrane integrity, and enzyme and protein stability (Chaves and Oliveira, 2004; Proietti et al., 2013) with possible positive effects on EVOO biochemical properties. While membrane integrity is essential to avoiding oil deterioration, the proteins present in the oil bodies of the mesocarp, which pass into the oil during the extraction process, contribute to its shelf life (Zamora et al., 2001).

Despite the importance of Se in EVOO in the Mediterranean diet, and in general for overall human health, there is still limited information concerning the effects of the micronutrient on olive oil quality. This present work has aimed to evaluate the effects of foliar Se fertilization on the antioxidant compounds that are involved in olive oil stability against oxidation, and, as a consequence, on human nutrition. Furthermore, since there is an increasing drought problem in the Mediterranean area, we have examined the effect of Se on EVOO obtained from both DS and well-watered olive trees.

## Materials and Methods

### Reagents and Standards

Sodium selenate (cod. S0882-25g), sodium sulfate anhydrous, and SeMet, Se-MeSeCys, and SeCys standards were purchased from Sigma-Aldrich (St. Louis, United States). The wetting agent Alba Milagro was provided by Alba Milagro International S.p.A (Milano, Italy). Acetone, n-hexane, water and methanol Optima grade were purchased from Fisher Scientific (Fair Lawn, NJ, United States). HNO_3_ (65% v/v), H_2_O_2_ (30% v/v), ultrapure grade HCl (37% v/v), methanol and mercaptoethanol were purchased from Carlo Erba Reagents (Milano, Italy).

### Experimental Setup

A 2-year field study (2014 and 2015) was conducted on a 17-year-old olive orchard (*O. europaea* L. cv Leccino) located at 43°02′ N, 12°43′ E with an altitude of 220 m (Central Italy). The field consisted of 18 rows, 78 m long, with a north-south orientation. There was a distance of 6.0 m between the rows and 3.0 m between each plant. In order to perform the experiment, two plots (one per year) with six rows per plot were selected. The olive trees, annually pruned, were trained to a monocone system (about 4 m high). Each spring, the olive orchard was fertilized using 16.5 t ha^-1^ of cow manure with the following composition: moisture 51.2%, organic matter 18.1%, total nitrogen (N) 0.68%, total phosphorus (P_2_O_5_) 0.30%, available potassium (K_2_O) 0.35%, available calcium (Ca) 0.70% and total Se 0.018 mg kg^-1^. The soil, derived from calcareous marl, was classified as Typic Haploxerept (Soil Survey Staff, 2014). It was initially characterized by a loam texture (sand 41%, silt 34%, clay 25%) with alkaline pH (8.1), and a concentration of 10.0 g kg^-1^ of total organic C, 2.0 g kg^-1^ of total N, 5.5 mg kg^-1^ of available P and 190 mg kg^-1^ of exchangeable K and 0.010 mg kg^-1^ of total Se. The location was characterized by a mean annual rainfall of 831 mm (1921–2015) with the wettest month being November (106 mm on average) and the driest one July (37 mm on average). The mean annual air temperature was 13.2°C (1951–2015), ranging from 23.2°C in July to 4.0°C in February. In 2014 the mean annual air temperature was 17.0°C, with August being the warmest month (27.7°C) and January the coldest one (7.5°C), while rainfall was 1074.6 mm (614 mm from April to October). In 2015 the mean annual air temperature was 17.0°C with August being the warmest month (28.1°C) and January the coldest one (7.2°C), while rainfall was 758.6 mm (377 mm from April to October). The environmental parameters of the study site in 2014 and 2015 have been reported in **Table 1**.

**Table 1:** Environmental parameters of the study site in 2014 and 2015.

	Years	
	
	2014	2015
Mean annual air temperature (°C)	17.0	17.0
Mean temperature in the warmest month (°C)	27.7 (August)	28.1 (August)
Mean temperature in the coldest month (°C)	7.5 (January)	7.2 (January)
Total annual rainfall (mm)	1074.6	758.6
Rainfall from April to October (mm)	614	377


For the DS trees no water was provided, while for the NDS trees irrigation was carried out in the morning, from late July to mid-September, using two drippers per tree, with a flow rate of 4 L h^-1^ each. With the aim of obtaining DS and NDS plants, each plot was divided into two sub-plots of three rows each. In 2014, the NDS trees were supplied with water at the rate of 0.19 m^3^ plant^-1^ in three interventions (2nd July, 28th July, and 10th August) for a total of 110 m^3^ ha^-1^, while in 2015 they were supplied with water at the rate of 0.26 m^3^ plant^-1^ in four interventions (4th July, 23rd July, 13th August, 25th August) for a total of 147 m^3^ ha^-1^. Irrigation was implemented when leaf water potential—measured with pressure chambers (Scholander chamber) reached -2 MPa (Gómez-del-Campo, 2013).

With the aim of obtaining Se fertilized (T) and NT plants under DS and NDS conditions, nine trees were Se-fertilized along each row in such a way that three T plants alternated with three NT ones. Between the T and NT treated trees there was one border tree. On 29th April each T tree was sprayed with 5 L per plant of a solution containing a Se concentration of 100 mg L^-1^, obtained by dissolving 239.26 g of sodium selenate in water, plus 0.5% of Albamilagro wetting agent.

### Plant Material Sampling, Yield, Fruit Ripeness Indexes and EVOO Extraction

To avoid any treatment interference, sampling was carried out on trees located in the center of each treatment area. As a consequence, three trees per treatment were selected (*n* = 3).

In both years of the study, at the beginning of November (harvest time), detachment force, flesh firmness, pigmentation, fresh and dry weight, and oil content were determined on 50 olives per tree, picked from the three selected plants per treatment.

Detachment force was measured using a Carpo hand dynamometer. Flesh firmness was determined by an Effe.gi dynamometer DT 05 with a 1.0 mm diameter tip. Fruit pigmentation was evaluated using the MI according to the Agronomic Station of Jaen method (Uceda, 2008) based on the evaluation of skin and pulp color.

The fresh and dry weight of the fruit was determined by weighing the olives before and after drying at 90°C for 48 h. Oil content was determined using a SpectraAlyzer ZEUTEC NIR: Near Infra Red.

Then, randomly selected fruit of three trees per treatment were harvested using pneumatic combs and net and weighed to estimate the yield.

Within 6 h of the harvest, 3.0 kg of olives from each sample were used to extract EVOO using a lab scale system. Fruit were crushed by a hammer mill the resulting paste was malaxed at 22°C for 20 min, and the oil separated by centrifugation. To remove water and impurities, the oil was filtered with cotton wool and sodium sulphate anhydrous, and then stored in glass bottles in the dark at 15°C until analysis.

### Preparation of Standard Solutions

Acid stock solutions of SeMet, SeMetSeCys, and SeCys standards, were prepared by dissolving the respective substances in 0.1 M HCl with 20% MeOH, except for SeMet, which was prepared in 0.5% 2-mercaptoethanol (0.3 mg g^-1^). Stock solutions were prepared once and stored at -20°C. Dilutions were made with 0.004% (m/v) aqueous solution of 2-mercaptoethanol to avoid oxidation of SeMet. Working standard solutions were prepared by appropriate dilution with ultrapure water, adjusted to pH with HCl or sodium hydroxide when it was required according to analysis.

### Oil Digestion and Protein Extraction

For total Se, 0.5 g of olive oil sample was treated with 7 mL concentrated HNO_3_ and 1 mL H_2_O_2_, and digested in a microwave oven (Milestone Inc., ETHOS One, Sorisole, Italy). Digestion was carried out at a ramp temperature of up to 200°C for 10 min, and a final hold time of 10 min. The employed microwave power was up to 1000 W. Total Se determination was performed with an ICP-MS (ELAN DRC-e; Perkin-Elmer SCIEX, Thornhill, Canada).

The modified method described by Martín-Hernández et al. (2008) was performed for protein extraction. 10 mL of cold n-hexane/acetone (1:1, v/v) (2°C) was added to 5 g of olive oil. The mixture was shaken vigorously, kept at 2°C for 1 h, and shaken every 10 min. The mixture was then centrifuged, and the supernatant was discarded. The precipitate was washed twice with 1 mL of cold n-hexane/acetone solution (1:1). After each washing, the mixture was centrifuged, and the supernatant was discarded. At both stages centrifugation lasted 10 min at 7000 rpm (6.026 × *g*) at 2°C in a refrigerated centrifuge [Boeco U-320 R; Boeckel + Co (GmbH + Co), Hamburg, Germany]. After the centrifugation stage, the supernatant was discarded and the pellet obtained was re-dissolved with water:methanol (80:20). This solution was centrifuged for 5 min at 3500 rpm (3.013 × *g*), followed by freezing at -18°C for 1 h to obtain a clear solution for analysis.

### EVOO Analysis

To achieve seleno-amino acid determination, the pellet obtained was treated for protein hydrolysis assisted by microwave (Reiz and Li, 2010), to this end 0.05% (v/v) phenol was added to avoid any amino acid oxidation by acids used during digestion. Mild conditions were used: 15% HCl (v/v) was added for a period of 5.5 min at a power of 900 W. Afterward, this solution was nitrogen evaporated at room temperature to avoid any volatilization of seleno-amino acids. The residue was then dissolved in 1 mL of 0.02 M HCl and filtered through a membrane filter before injection (200 μL) on LC–ICP MS. Seleno-amino acid determination was performed by coupling the chromatographer (Series 200; Perkin-Elmer, Thornhill, Canada) to ICP MS. Hydrolyzed fractions were analyzed for seleno-amino acids using reverse phase chromatography (RPC). The selected isotope for mass monitoring by ICP-MS was ^82^Se, in order to avoid interference by polyatomic argon. The RPC–ICP MS conditions for separation of seleno-species by reverse phase chromatography are summed up in **Supplementary Table S1**.

### Total Phenol, Carotenoid and Chlorophyll Contents, and Oxidative Stability

Phenol content was determined using the Folin-Ciocalteu reagent according to the Folin–Ciocalteu method (Singleton et al., 1998) and the results were expressed as μg gallic acid equivalent g^-1^ oil.

Total chlorophyll and total carotenoids were extracted dissolving 0.5 g of oil sample in 25 mL of 95% diethyl ether. The solution was filtered through a double layer of cheese cloths and the absorbance of the extract was measured with a Varian Cary 210 spectrophotometer at 662, 646, and 470 nm (Lichtenthaler and Wellburn, 1983).

The stability against oxidation, expressed as the oxidation induction time (hours), was estimated with a Rancimat 679 apparatus (Metrohm Co., Herisau, Switzerland) using 5.0 g of oil heated at 120°C with an air flow of 20 L h^-1^ passing through the sample. The volatile compounds were collected in a conductivity cell filled with distilled water. The time needed for the appearance of a sudden water conductivity rise caused by the adsorption of volatiles derived from the oil oxidation was registered as the induction time in hours.

### Statistical Analysis

A two-way ANOVA was conducted to analyze the effects of Se fertilization and irrigation on the selected EVOO parameters. The assumption of normality and homoscedasticity of the data was verified by graphical analysis of residuals, and transformed where necessary. Means were compared to Tukey’s *post hoc* test (*P* < 0.05) and the statistical analyses were performed using R software (R Development Core Team, 2011).

## Results

### Ripening Indexes and Production of Olives

While in 2014 no differences were observed between the treatments, in 2015 a higher fruit yield and detachment force, and lower pigmentation was found in the NDS plants than in the DS plants (**Table 2**). Furthermore, in 2015 the T trees under drought stress showed a higher fresh weight fruit yield than the NT ones.

**Table 2:** Fruit yield tree^-1^ expressed both as fresh and dry weights, color, pulp firmness, detachment force and oil content of olive (cv Leccino) orchard fertilized (T) and unfertilized (NT) with Se under well-watered (NDS) and drought stress (DS) conditions.

Year	Irrigation	Treatment	Fruit yield tree^-1^		Color	Pulp firmness	Detachment force	Oil content
							
			kg fresh weight	kg dry weight	0–7	N	N	%
2014	DS	NT	23.2 (1.2)a	11.0 (0.5)a	4.4 (0.3)a	3.4 (0.2)a	4.6 (0.4)a	17.8 (1.6)a
2014	DS	T	22.4 (1.0)a	10.6 (0.8)a	5.0 (0.3)a	3.7 (0.3)a	4.6 (0.1)a	17.5 (1.5)a
2014	NDS	NT	21.8 (1.1)a	10.5 (0.7)a	4.6 (0.4)a	3.4 (0.1)a	4.4 (0.2)a	17.4 (1.4)a
2014	NDS	T	21.2 (0.9)a	11.0 (0.6)a	4.5 (0.3)a	3.1 (0.3)a	4.6 (0.6)a	17.9 (1.4)a
2015	DS	NT	28.8 (0.8)c	17.7 (0.9)b	4.8 (0.1)a	4.0 (0.2)a	4.7 (0.2)b	16.9 (1.6)a
2015	DS	T	33.6 (1.2)b	18.6 (1.1)b	5.0 (0.2)a	3.9 (0.2)a	4.8 (0.3)b	17.3 (1.3)a
2015	NDS	NT	38.1 (1.1)a	20.7 (0.7)a	3.5 (0.1)b	4.8 (0.2)a	5.8 (0.4)a	17.0 (1.7)a
2015	NDS	T	37.7 (0.9)a	20.6 (0.9)a	3.9 (0.1)b	4.9 (0.1)a	5.9 (0.3)a	17.0 (1.4)a


*Values are means with SEs in parentheses (*n* = 3). In each column and for each year, different letters indicate significant differences among treatments at *P* < 0.05 using Tukey-HSD test.*

### Carotenoid and Chlorophyll Content, and Chlorophyll-to-Carotenoid Ratio of EVOO

Whereas Se application increased EVOO carotenoid content under both drought stress and well-watered conditions, a higher carotenoid concentration was observed in the EVOO obtained from the NT trees under DS than under NDS conditions (**Table 3**). Unlike carotenoids, the EVOO chlorophyll content was lower under NDS compared to DS condition, but similarly to carotenoids, Se application increased their amount in the EVOO (**Table 3**).

**Table 3:** Carotenoids, chlorophylls, phenols contents, chlorophylls-to-carotenoids ratio (Chlor/Carot), and stability against oxidation (Stability) in extra virgin olive oils obtained from olive (cv Leccino) orchard fertilized (T) and unfertilized (NT) with Se under well-watered (NDS) and drought stress (DS) conditions.

Year	Irrigation	Treatment	Carotenoids	Chlorophylls	Phenols	Stability	Chlor/Carot
					
			μg g^-1^			h	
2014	DS	NT	10.6 (0.3)b	14.2 (0.2)c	287 (6)b	12.4 (0.2)b	1.34 (0.04)bc
2014	DS	T	13.0 (0.2)a	19.6 (0.3)a	343 (9)a	14.3 (0.2)a	1.52 (0.04)b
2014	NDS	NT	7.7 (0.1)c	14.3 (0.3)c	257 (7)c	11.0 (0.3)c	1.87 (0.05)a
2014	NDS	T	13.7 (0.4)a	15.8 (0.4)b	274 (4)bc	11.7 (0.2)bc	1.16 (0.05)c
2015	DS	NT	11.7 (0.3)c	15.6 (0.2)c	246 (5)c	11.8 (0.1)b	1.34 (0.03)b
2015	DS	T	14.0 (0.3)b	21.7 (0.3)a	296 (4)a	13.6 (0.1)a	1.55 (0.01)a
2015	NDS	NT	9.2 (0.1)c	14.5 (0.2)d	245 (6)c	10.0 (0.1)c	1.58 (0.02)a
2015	NDS	T	15.5 (0.4)a	18.5 (0.2)b	262 (3)b	11.1 (0.2)b	1.19 (0.02)c


*Values are means with SEs in parentheses (*n* = 3). In each column and for each year, different letters indicate significant differences among treatments at *P* < 0.05 using Tukey-HSD test.*

In both years, while under drought stress the EVOO chlorophyll-to-carotenoid ratio was increased with Se application, conversely, in well-watered conditions the ratio decreased with Se application (**Table 3**). Furthermore, while in NT samples the chlorophyll-to-carotenoid ratio increased with irrigation, the opposite was observed in EVOO obtained from T trees.

### Phenol Content and Stability Against Oxidation of EVOO

The promoting effect of Se treatment to EVOO phenol content and stability against oxidation was more pronounced under DS compared to NDS conditions, especially in the second year.

### Total Se, SeMet, SeCys, and SeMetSeCys Contents of EVOO

In both years, while Se application increased the Se concentration in EVOO samples, irrigation reduced its concentration (**Table 4**).

**Table 4:** Total Se, SeCys, SeMet, and SeMeSeCys contents in extra virgin olive oils obtained from olive (*Olea europaea* L. cv Leccino) orchard fertilized (T) and unfertilized (NT) with Se under well-watered (NDS) and drought stress (DS) conditions.

Year	Irrigation	Treatment	Total Se	SeCys	SeMet	SeMetSeCys
			
			μg kg^-1^			
2014	DS	NT	149 (9)c	n.d.	n.d.	n.d.
2014	DS	T	378 (8)a	n.d.	4.83 (0.14)	n.d.
2014	NDS	NT	16 (1)d	n.d.	n.d.	n.d.
2014	NDS	T	189 (6)b	n.d.	n.d.	n.d.
2015	DS	NT	164 (8)b	n.d.	n.d.	n.d.
2015	DS	T	529 (7)a	n.d.	4.17 (0.11)b	n.d.
2015	NDS	NT	12 (1)c	n.d.	n.d.	n.d.
2015	NDS	T	171 (6)b	n.d.	5.39 (0.06)a	n.d.


*Values are means with SEs in parentheses (*n* = 3). In each column and for each year, different letters indicate significant differences among treatments at *P* < 0.05 using Tukey-HSD test.*

*n.d., not detected.*

While SeCys and SeMetSeCys were not detected in any olive oil samples, SeMet was found in all samples obtained from T trees with the exception of the EVOO obtained from the NDS plants in 2014. In addition, in 2015 a higher SeMet content was found under NDS than under DS conditions (**Table 4**).

## Discussion

The weather conditions during the two growing seasons were quite different. Compared to the 1921–2015 period, the mean annual rainfall in 2014 was +29%, whereas in 2015 it was -11%.

In 2015, according to Proietti et al. (2013) and D’Amato et al. (2014), Se treatments reduced the negative effects of low water availability on the olive yield per tree. In fact, because of its stimulating effect on root water uptake, Se increased the tolerance of plants to drought stress by regulating the water status (Djanaguiraman et al., 2005). The lower pigmentation and higher detachment force and pulp firmness of olive drupes from the NDS trees as compared to those from the DS ones might be due to the higher fruit yield (crop load) induced by irrigation. This fact might have caused a slowing down in fruit ripening due to a reduced availability of assimilates per drupe (Proietti et al., 2013). The absence of any differences in 2014 could be attributed to the more abundant rainfall and to a lower crop load (an off year) which possibly masked the DS effect. Conversely, Se treatment did not influence the fruit ripening process.

As expected, the foliar application of Se increased the Se content in EVOO samples. These results are consistent with previous studies which, after foliar Se treatments, found enhanced Se concentrations in several agricultural crops such as rice (Li et al., 2008), winter wheat (Ducsay and Ložek, 2006), carrot (Kápolna et al., 2009), peach and pear (Pezzarossa et al., 2012) as well as in agri-food industry products such as EVOO and wine (D’Amato et al., 2014; Fontanella et al., 2017). The higher quantities of total Se in EVOO obtained from DS plants than from irrigated ones, might well be attributed to the ability of Se to increase the drought stress tolerance of plants (Proietti et al., 2013). The ability of Se to improve photosynthesis and protect PSII in fruit crops (peach and pear) was reported also by Feng et al. (2015). Since Se has this positive effect on plants that are exposed to stress conditions, there might be the risk of the plants tending to accumulate Se. The findings of this study are in accordance to those of Nawaz et al. (2016), which found a higher total Se content in wheat shoots grown under drought stress conditions, than wheat shoots grown under well-watered conditions. However, because of the high water solubility of Se, we cannot exclude a possible higher loss of Se during the EVOO extraction process in the NDS olive drupes, than in the DS ones.

The higher carotenoid and chlorophyll contents in the T treatments than in the NT ones might indicate the stimulatory effect of Se on the biosynthesis of the photosynthetic pigments (Malik et al., 2012; Hashem et al., 2013). This aspect is interesting in relation to the EVOO stability against oxidation and to its nutritional value. Indeed, while in EVOO carotenoids and chlorophylls play an important role in oxidative processes due to their antioxidant nature in the dark, and their pro-oxidant activity in the light (Tovar et al., 2002), in humans carotenoids prevent cardio-vascular disease, show anti-cancer activity and provide protection against UV radiation (Tapiero et al., 2004). In contrast to Gómez-Rico et al. (2007) and Tovar et al. (2002), the carotenoid concentration of EVOO obtained from the NT trees decreased with irrigation, which may be due to the lowered stress conditions of the irrigated plants (Munné-Bosch and Alegre, 2000). In fact, it is well known that plants tend to accumulate carotenoids when exposed to some stress factors such as drought (Reddy et al., 2004). However, we cannot exclude that the lower content of carotenoids, as well as chlorophylls, might be due to the delay of the fruit ripening processes (Beltrán et al., 2005; D’Amato et al., 2014; Nasini and Proietti, 2014). For the T treatments, the lack of differences in carotenoid concentration between NDS and DS plants might be due the role of Se in alleviating the drought stress conditions (Nawaz et al., 2015).

Since changes of the chlorophyll-to-carotenoid ratio can indicate water deficient conditions (Jaleel et al., 2009; Ramakrishna and Ravishankar, 2011), the higher ratio in EVOO from the NDS-NT group as compared to the DS-NT one, and from the DS-T group as compared to the DS-NT one, might confirm the capacity of Se to protect plants from drought stress damage (Hasanuzzam et al., 2010; Proietti et al., 2013; Ahmad et al., 2016). In fact, Se has protective effects on chloroplast enzymes with a consequent increase in photosynthetic pigment biosynthesis (Pennanen et al., 2002). However, the higher chlorophyll-to-carotenoid ratio could be also partly attributed to the delay in the ripening process, due to a higher yield per tree (Nasini et al., 2013).

Under well-watered conditions, the lower chlorophyll-to-carotenoid ratio of EVOO from NT plants compared to those from T ones could be due to an inhibitory effect of Se on the production of an enzyme involved in chlorophyll biosynthesis (Fargašová et al., 2006).

The lower phenol content in EVOO from irrigated rather than non-irrigated trees could be attributed to the different water availability. Indeed, it is well-known that the level of phenols is higher in oil from drought-stressed plants than from irrigated ones (Jose Motilva et al., 2000; Servili et al., 2007). Furthermore, as already reported by D’Amato et al. (2014), Se treatment increases EVOO phenol content. However, the higher levels of phenols in EVOO from Se-treated trees may be due to a secondary effect caused by the inhibition of enzymatic phenol oxidation by strong antioxidant-active Se compounds (D’Amato et al., 2014). Since the high phenol content in EVOO could be considered a positive aspect because the higher the phenol content, the greater the oxidative stability, the increase of phenol content obtained from the DS and T treatments resulted in an increase of EVOO oxidative stability (Tovar et al., 2002; Ayton et al., 2007; Gouveia et al., 2013).

The higher stability in the T than in the NT EVOO could be also due to the greater content of both chlorophyll and SeMet which are involved in the anti-oxidative processes that take place in the olive oil (Zalejska-Fiolka, 2000; Vacca et al., 2006). The enhanced amount of Se in the form of SeMet could be interesting for both human health and olive oil quality. Indeed, while in humans SeMet is the only Se amino acid that can form proteins and provide several positive effects on human health (Schrauzer, 2000; Navarro-Alarcon and Cabrera-Vique, 2008) in EVOO SeMet is involved in the anti-oxidative processes (Zalejska-Fiolka, 2000; Vacca et al., 2006) which promote its stability.

Our findings are in contrast to Torres et al. (2004) who found the presence of SeMeSeCys in seven EVOO samples from different Argentinian regions. In the present study only selenomethionine has been detected in EVOO from Se fertilized plants.

Ultimately, Se-biofortification may well be of interest to areas with poor cultivars or cold, rainy weather patterns, which would normally lead to the production of EVOO with an unfavorable phenol content.

## Conclusion

Olive tree (*O. europaea* L.) is the most important evergreen tree in the Mediterranean basin and EVOO is regarded as a key component of the traditional Mediterranean diet. The present study demonstrated that Se fertilization through foliar application can be adopted in olive groves to improve some qualitative and nutritional properties of EVOO. Indeed, our findings suggested how Se application, besides to enhance the Se content, increased the concentration of the oil antioxidant compounds. The greater amounts of these molecules, such as chlorophylls, carotenoids, phenols, and SeMet, brings advantages for the EVOO itself because enable to increase the EVOO oxidative stability and, as consequence, its shelf-life. Furthermore, in a drought scenario, this work confirmed the important role of Se to alleviate the negative effect of water deficiency on fruit production and olive oil stability against oxidation. As a consequence, this study highlighted how Se fertilization could be a suitable technique in areas characterized by low Se contents and subjected to frequent drought events to obtain high quality EVOOs.

## Author Contributions

PP, LR, and RD designed the experiments. PHP, PH, RD, and LR performed the analytical assays and analyzed the data. PP, AO, MDF, RD, DB, and LR wrote the manuscript. PP, DB, MDF, RD, LR, and AO revised the manuscript.

## Conflict of Interest Statement

The authors declare that the research was conducted in the absence of any commercial or financial relationships that could be construed as a potential conflict of interest.

## Abbreviations

ANOVA: analysis of variance
DS: drought stressed
EVOO: extra-virgin olive oil
GPx: glutathione peroxidase
HCl: hydrochloric acid
HNO_3_: nitric acide
H_2_O_2_: hydrogen peroxide
LC–ICP MS: liquid chromatography–inductively coupled plasma mass spectrometry
MI: maturity index
MeOH: methanol
NDS: non-drought stressed
NT: not Se-treated plants
ROS: reactive oxygen species
RPC–ICP MS: reversed-phase chromatography inductively coupled plasma mass spectrometry
Se: selenium
SeCys: selenocysteine
SeMeSeCys: selenomethylselenocysteine
SeMet: selenomethionine
T: Se-treated plants.
