# Supplementary material for: The Selenium Supplementation Influences Olive Tree Production and Oil Stability Against Oxidation and Can Alleviate the Water Deficiency Effects
Source: Front Plant Sci. 2018 Aug 15;9:1191. doi: 10.3389/fpls.2018.01191 (PMC6104596; doi:10.3389/fpls.2018.01191)
Supplement: Supplementary file 1 [file Table_1.DOCX]

**Table S1.** LC–ICP-MS conditions for selenium distribution analysis.

| **Chromatographic conditions** | |
| --- | --- |
| *Reversed Phase Chromatography* | |
| Stationary phase | Agilent Zorbax SB C18 (4.6 mm x 15 cm x 5µm) |
| Mobile phase | 98:2 water–methanol, 10mM TFA pH 3.0 |
| Flow rate | 1.0 mL min^−1^ |
| Injection volume | 200 µL |
| *Size Exclusion Chromatography* | |
| Mobile phase | Ammonium acetate 50 mM, 5% methanol (v v^-1^) |
| Elution mode | Isocratic |
| Flow rate | 0.9 mL min^-1^ |
| Column | TSK gel G3000SW (7.5mm x 300 mm x 10 µm) |
| Sample Loop | 200 µL |
| **ICP MS conditions** | |
| RF Forward power | 1050 W |
| Gas flow rates: | |
| Plasma | 13 min^−1^ |
| Auxiliar | 1.35 min^−1^ |
| Nebulizer | 0.75 min^−1^ |
| Resolution | Normal |
| Scaning mode | Peak hop |
| Dwell time | 500 ms |
| Isotope monitored | Se^82^ (with Krypton correction) |
